# Accumulation and Elimination of Tetrodotoxin in the Pufferfish *Takifugu obscurus* by Dietary Administration of the Wild Toxic Gastropod *Nassarius semiplicata*

**DOI:** 10.3390/toxins12050278

**Published:** 2020-04-25

**Authors:** Xiaojun Zhang, Jingjing Zong, Si Chen, Menglong Li, Yibo Lu, Ruirui Wang, Hanxiang Xu

**Affiliations:** 1 Laboratory of Aquatic Product Processing and Quality Safety, Marine Fisheries Research Institute of Zhejiang, Zhoushan 316100, China; zhangxj@zjou.edu.cn (X.Z.); sichen_ns@zjou.edu.cn (S.C.); 2 Zhejiang Province Key Lab of Mariculture & Enhancement, Zhoushan 316100, China; 3 School of Food and Pharmacy, Zhejiang Ocean University, Zhoushan 316022, China; zongjingjing@hotmail.com (J.Z.); luyibo123@gmail.com (Y.L.); wangrui0910@hotmail.com (R.W.); 4 Quality and Standard Research Center, Chinese Academy of Fishery Sciences, Beijing 100141, China; liml@cafs.ac.cn

**Keywords:** tetrodotoxin, *Takifugu obscurus*, accumulation, elimination, *Nassarius semiplicata*

## Abstract

To investigate pufferfish accumulation, elimination, and distribution of tetrodotoxin (TTX), *Takifugu obscurus* was fed with wild TTX-containing gastropod *Nassarius semiplicata* to simulate the natural food chain. Three-month-old non-poisonous *T. obscurus* was fed with wild toxic *N. semiplicata* at three exposure dose for 28 days, and later, with toxin-free food until day 67. Three fish individuals from each treatment were sampled, and the distribution of TTX in different tissues was measured. The results showed that the accumulation ratio of TTX in the three exposure dose groups ranged from 35.76% to 40.20%. The accumulation ratio in the skin and liver was the highest amongst all tissues, accounting for more than 85% of the total TTX, whereas that in the kidney and gallbladder was the lowest (0.11–0.78%). Studies on the kinetic of TTX accumulation and elimination revealed that the skin was the tissue with the highest accumulation speed constant (8.06), while the liver, kidney, and intestinal tract showed the highest speed of TTX elimination. The time required for TTX reduction to reach the safety limit could be predicted by using standard elimination equations. Qualitative analysis by UPLC-MS/MS revealed the occurrence of seven TTX derivatives in *T. obscurus*; of these TTX, 5-deoxy TTX, 11-deoxy TTX, 4,9-anhydro TTX were found in all tested tissues.

## 1. Introduction

Tetrodotoxin (TTX) is a heterocyclic organic perhydroquinazoline compound that was initially isolated from wild *Tetraodontidae* by Tahara in 1909 [1,2,3,4]. TTX has the chemical formula of C_11_H_17_O_8_N_3_ and a low molecular weight of 319 [5]. TTX, mainly found in pufferfish, is a chemical with high stability. As a potent non-protein neurotoxin, TTX can selectively block sodium channels to inhibit nerve and muscle conductions, leading to death from nerve paralysis and respiratory failure [6,7,8]. The median lethal dose (LD50) for TTX found after intraperitoneal injection in mice was 10.7 μg/kg [9,10]. In the case of human intoxication, the onset of symptoms could occur within 10 min after ingestion, with a delay of up to 4–6 h. No effective antidote for TTX has been reported yet. Pufferfish is a delicacy appreciated in East Asia. In recent years, there have been cases of poisoning and even death caused by eating poisonous pufferfish [10,11,12,13,14,15,16]. To maintain seafood safety and a healthy development of the pufferfish industry, health authorities in Japan have set an acceptable limit of TTX of < 10 MU/g in edible parts of pufferfish [16] and a regulatory limit in seafood of 2000 μg/kg [9,10]. There are no food regulations concerning TTX in the United States of America [17].

Previous studies have suggested that TTX may be initially synthesized by marine bacteria and transferred to pufferfish via the food chain [2,18]. Nevertheless, the mechanisms of TTX accumulation in the pufferfish through a toxic food chain, and its elimination remains to be elucidated [19]. It has been shown that cultured non-toxic pufferfish when fed on a TTX-containing diet become toxic, and TTX mainly accumulated into the liver and skin [20], while pufferfish should become non-toxic when fed TTX-free diets after hatching in an environment in which the invasion of TTX-bearing organisms has been eliminated [21]. Furthermore, TTX accumulation and transfer kinetics in *Takifugu rubripes* [22], *Takifugu porphyreus* [23]*, Takifugu poecilonotus* [24], *Takifugu niphobles* (current name: *Takifugu alboplumbeus*) [20] and other pufferfish [25] indicated that the distribution, accumulation, and elimination of TTX varied among different species. 

In 2016, China allowed certified companies to cultivate pufferfish *T. rubripes* and *T. obscurus*. The accumulation and elimination patterns of TTX in *T. rubripes* have been extensively studied [19]. TTX mainly accumulated in the skin, liver, and ovary [20], but there were important differences according to the age group: TTX accumulated mainly in the liver in adult fish, whereas in juvenile fish the maximum was found in the skin [19,26,27]. Study on the accumulation, elimination, and distribution of TTX in *T. obscurus* has not been reported, yet the related information is essential to prevent and control the occurrence of TTX poisoning.

In this study, *T. obscurus* was used as a model organism to simulate the accumulation and elimination of TTX in the natural food chain by feeding it with wild TTX-containing marine snail *Nassarius semiplicata*. The study aims to describe the temporal–spatial patterns of TTX distribution in different tissues by analyzing time–tissue data, thereby understanding the accumulation and elimination patterns of TTX in *T. obscurus* under natural feeding conditions.

## 2. Results

### 2.1. Anatomical Distribution of TTX

The accumulation of TTX by *T. obscurus* was evaluated by comparing the actual intake and feeding amount at the end of the accumulation period. Total TTX content in samples from the three exposure dose groups was determined on day 28, and the actual intake, actual accumulation, and accumulation ratio of TTX were calculated and recorded (Table 1). TTX intake ranged from 1032 to 5420 ng, and the TTX accumulation ratio from 35.76% to 40.20%. The actual feed intake of different dose groups was similar, and the actual TTX accumulation gradually increased with the dose concentration. Unlike the total accumulation pattern, the TTX accumulation rate did not increase with the increase in intake but showed a unique pattern. The accumulation ratio of the low-dose group was the highest, reaching a value of 40.20%, whereas that of the medium-dose group (35.76%) was the lowest. The above results showed that TTX could be accumulated more efficiently in *T. obscurus* under low-dose exposure, but there was no linear relationship between TTX accumulation and the exposure dose.

**Figure 1:**
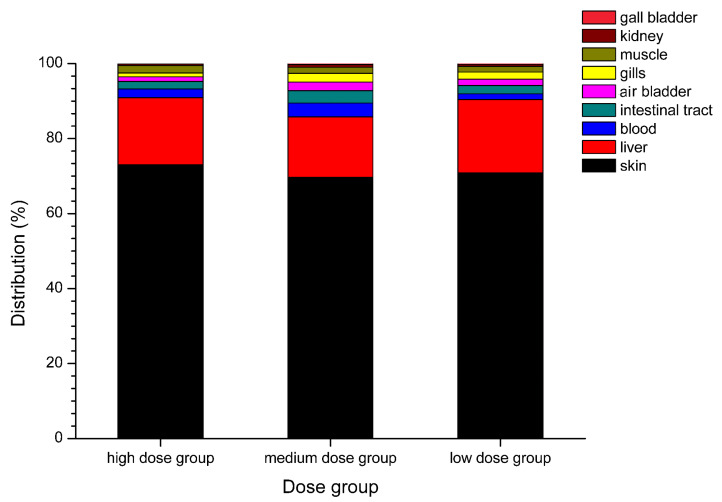
Anatomic distribution of tetrodotoxin (TTX) in *Takifugu obscurus* under low-dose (100 ng TTX/g), medium-dose (210 ng TTX/g), and high-dose (600 ng TTX/g) feeding conditions.

Various tissues of *T. obscurus* were collected on day 28 at the end of the accumulation stage to measure TTX concentration by UPLC-MS/MS. TTX distribution was subsequently analyzed by combining data of TTX concentration with tissue weight. The proportion of the accumulated amount of TTX in each tissue with respect to content is shown in Figure 1. Overall, the TTX amount in the skin accounts for the largest proportion (69.70–73.00%), followed by the liver (16.05–19.58%). These two tissues account for more than 85% of the total amount of TTX. The TTX amount in the blood, intestinal tract, air bladder, gills, and muscle was relatively low, proportion values ranging from 3.72% to 0.99%. The TTX proportion in the kidney and gallbladder was the lowest, accounting for only 0.11–0.78%. Differences in TTX distribution were also observed among different dose groups. The TTX proportion in the blood from the medium- and high-dose groups was higher than that in the intestine, air bladder, gills, and muscle, while the TTX proportion in the blood from the low-dose group was significantly lower.

### 2.2. Patterns in TTX Accumulation and Elimination

**Figure 2:**
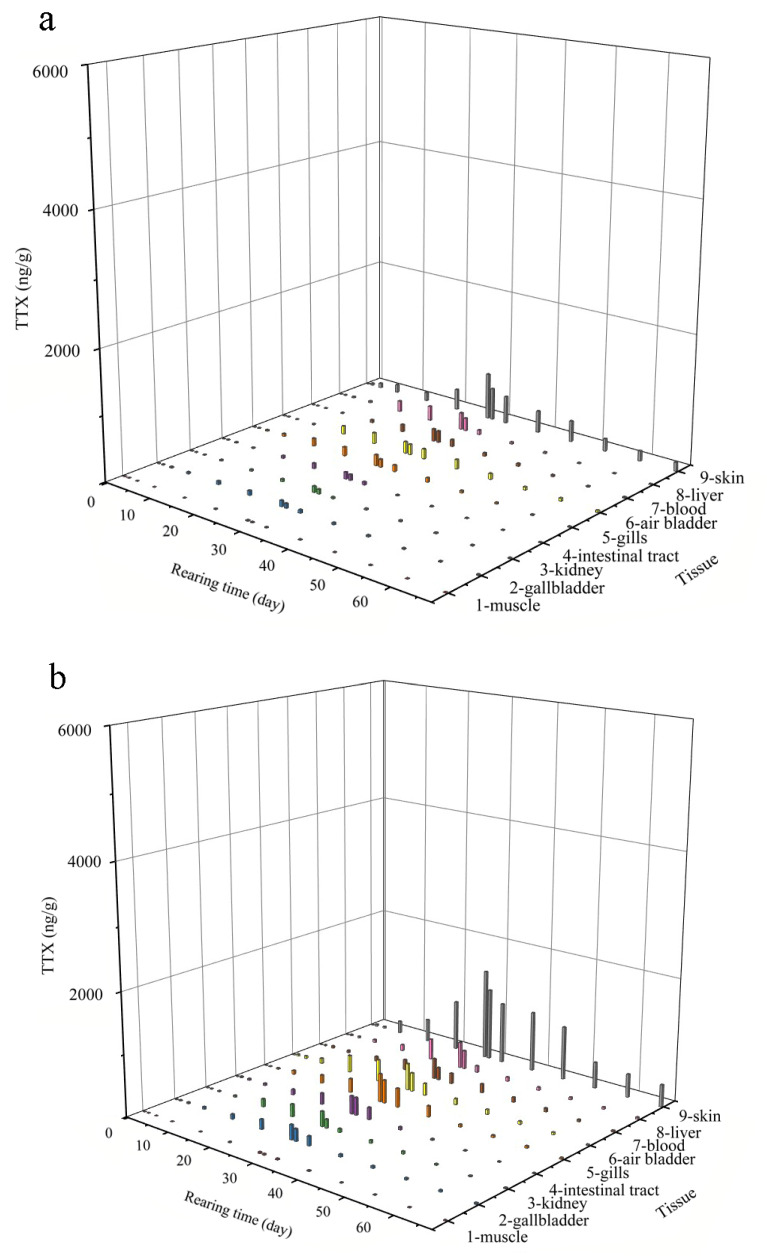

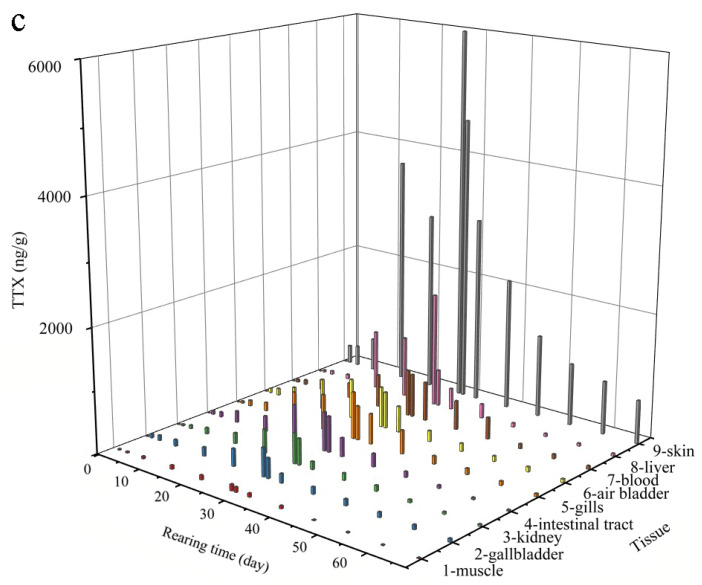
Patterns of TTX accumulation and elimination in different tissues of *T. obscurus* under low-dose (100 ng TTX/g), medium-dose (210 ng TTX/g), and high-dose (600 ng TTX/g) feeding conditions. (**a**) low-dose. (**b**) medium-dose. (**c**) high-dose.

To systematically study the accumulation and elimination pattern in *T. obscurus*, various tissues were collected at different time points during the accumulation and elimination period to determine the TTX concentrations, and the data were analyzed to obtain a 3D change diagram of time-tissue-concentration (Figure 2). The TTX concentration first increased and then decreased with time throughout the accumulation and elimination process, showing a Gaussian distribution. The accumulation and elimination of TTX in different tissues were significantly different.

During the 28-day accumulation period, TTX levels in of the three exposure dose groups of *T. obscurus* increased with the number of toxic feeding days. In all tissues, the initial accumulation rates were relatively slow (day 1–7), ranging from 0.17 ng/g/d to 39.20 ng/g/d. The accumulation rates were the fastest in the middle period (day 7–21), rising from 0.86 ng/g/d to 338.09 ng/g/d. In the later stage (day 21–28), the accumulation rates gradually slowed down, dropping from 0.33 ng/g/d to 328.57 ng/g/d. At the end of the accumulation stage (day 28), the TTX concentration in the skin reached the highest values (Figure 2), with 717.4, 1484, and 6000 ng/g in the low-, medium- and high-dose groups, respectively. For the liver tissue, values were 262.2, 427.6, and 1840 ng/g, respectively, for muscle tissue, the values were 10.68, 20.51, and 99.37 ng/g. To evaluate the accumulation in *T. obscurus*, the TTX accumulation rate constant was calculated by using the relationship between the accumulation concentration and feeding concentration. The variance analysis showed that there was no significant difference (*p* > 0.05) in the accumulation rate constant of the same tissue among three exposure doses. Different tissues were divided into four groups according to the value of their accumulation rate constant: (i) rapid-constant group (skin tissue) with a maximum of 8.06; (ii) sub-rapid-constant group, including the liver (2.58), air bladder (1.74), gills (1.62), and blood (1.58); (iii) medium-rapid-constant group, including the intestine (1.15), kidney (0.96), and gallbladder (0.93), and (iv) low-constant group, including muscle tissue, with a minimum value of 0.12.

During the elimination period, the TTX content in the tissues showed a downward pattern, with the fastest decline from the 29th to the 39th day, and a much slower in the later period (day 39–67). To gain a better understanding of the characteristics of TTX elimination, the kinetics of toxin elimination in different tissues was studied (Table 2). The elimination rate constant K could directly reflect the elimination rate of the toxin. Variance analysis revealed there were no significant differences in the elimination rate constant K (*p* < 0.05) among different dose groups using variance analysis, which indicated that different dosages had little effect on the elimination rate, leading to a very similar elimination process. This pattern can also be visualized in Figure 2. In terms of different tissues, the elimination rates were obviously different. The tissues with the highest clearance rates were the liver, kidney, and intestinal tract, and their K values were 0.952, 0.496, and 0.236, respectively. The tissues with lower elimination rates included the gallbladder, air bladder, and gills, with K values of 0.132, 0.138, and 0.100, respectively. The tissues with the lowest rate of elimination were the gallbladder, muscle, and skin, with the K values of 0.059, 0.040, and 0.035, respectively. There were also obvious differences in the total elimination of TTX in different tissues. After 39 days of elimination experiment, the residual TTX content in the muscle from the low-, medium-, and high-dose feeding groups were all found to be the lowest, reaching 2.32 ng/g, 2.79 ng/g, and 7.4 ng/g, respectively. Skin tissues of the three groups with values of 135.66, 358.79, and 699.35 ng/g, respectively, contained the highest content of residual TTX. TTX in the skin reached a maximum concentration of 6000 ng/g, which is three times the regulatory limit at the end of accumulation (Table 2). By substituting the concentration into the elimination model, it is estimated that TTX in the skin could be below the safety limit after 12.3 days.

### 2.3. TTX Derivatives in Takifugu obscurus

**Figure 3:**
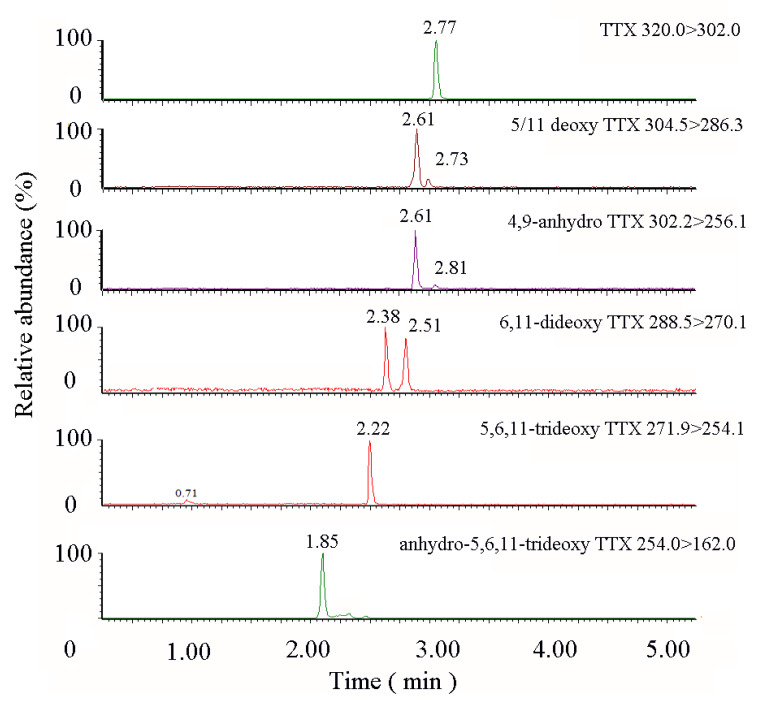
UPLC-MS/MS Chromatogram of TTX derivatives in *Takifugu obscurus.*

To know whether TTX is transformed or degraded in *T. obscurus* during the accumulation period, TTX derivatives in the feed and the pufferfish after the enrichment experiment were analyzed. The results showed that only TTX was detected in *Nassarius semiplicata*, while a variety of TTX derivatives were found in different pufferfish tissues, as shown in Table 3. TTX, 5-deoxy TTX, 11-deoxy TTX, 4,9-anhydro TTX were found in all tissues, indicating the common existence of the above substances. While anhydro-5,6,11-trideoxy TTX was only detected in the skin. The UPLC-MS/MS chromatogram of TTX derivatives in *T. obscurus* is shown in Figure 3. 

## 3. Discussion

A TTX exposure model of *T. obscurus* cultured in brackish water was established. The pufferfish was fed with wild *Nassarius semiplicata* to simulate the natural food chain environment. Differences in the anatomical distribution of TTX have been observed between marine and freshwater pufferfish [10,25], but also between juvenile and mature individuals [22]. Here we studied detection and anatomical distribution of TTX in 3-month-old *T. obscurus*, which is the least studied freshwater species. There is a variety of TTX administration modes. In most studies, TTX administration mode is in the form of intramuscular administration (IMA) [26], in which a one-time injection cannot reflect the real accumulation process. Oral gavage administration (OGA) was used in other studies [22,23], but the passive acceptance process cannot reflect the real natural accumulation. In this study, TTX-containing *N. semiplicata* was chosen as the 28-day feeding source to get TTX-containing *T. obscurus*, followed by 36-day of observations on the elimination process. These conditions simulated well the natural food chain.

The pufferfish *T. obscurus* could accumulate TTX efficiently with an accumulation ratio of 35.76–40.20%. The ability to accumulate toxins varies among different organisms. Pufferfish, mussel, starfish, and salamander have proven to be capable of accumulating biotoxins. Among them, pufferfishes are the most common TTX-accumulating species [2]. The capacity to accumulate TTX in different pufferfish species is obviously different. Wang et al. [23] performed an accumulation experiment with the artificial hybrid of *Takifugu rubripes* and *Takifugu porphyreus* using gastric gavage and intramuscular injection and found that the accumulated amount of TTX in the above groups ranged from 31% to 45% and from 42% to 74%, respectively. Tatsuno et al. [22] fed a TTX-containing homogenate to non-toxic cultured *T. rubripes* by gastric gavage and the total amount of TTX accumulated per fish was 31% of the given dose. In this study, *T. obscurus* was fed with poisonous wild *N. semiplicata* to mimic the predator’s behavior in the field. The average amount of TTX accumulated in *T. obscurus* was 38.03–40.20%, which is similar to that obtained from the gastric gavage method [23], but significantly lower than that obtained from the intramuscular injection method. Gao et al. [25] compared the selective accumulation of TTX between freshwater and marine pufferfish and found that freshwater *P. suvatti* could only accumulate a very small amount of TTX (0.03–0.5%). As brackish-water pufferfish, *T. obscurus* showed much higher TTX accumulation capacity in this study.

We also found that the distribution of TTX in *T. obscurus* was tissue-specific. Noguchi et al. [2] demonstrated that in adult fish, the liver and ovaries usually contained the highest toxin content, followed by the intestine and skin. Tatsuno et al. [22] compared TTX accumulation in the juvenile *T. rubripes* (6 and 15 months old). The 6-month-old pufferfish had the highest content of TTX accumulated in the skin (71%), followed by the liver (21%). For the 15-month-old pufferfish, the amount of TTX was highest in the liver (83%), and that in the skin was 14%. *T. obscurus* specimens selected for the current study were 3-month old, so their ovary development was still incomplete. UPLC-MS/MS analysis of the juvenile *T. obscurus* revealed that the TTX content in the skin (69.35–73.00%) was significantly higher than that in the liver (16.05–19.16%), which was similar to the reported values in 6-month-old *T. rubripes*. Itoi et al. [28,29] observed that non-toxic fish would rapidly spit out juvenile pufferfish (*T. rubripes* and *T. niphobles*) after swallowing them. Therefore, it can be assumed that the high accumulation of TTX in the skin of *T. obscurus* is also a self-protection adaption to repel predators in nature.

For the high-dose group, the TTX concentration in the skin decreased on the 21st day (Figure 2c), while in the medium- and low-concentration groups, the TTX concentration increased gradually with time during the whole accumulation period (Figure 2a,b). This phenomenon underlines the fact that the TTX accumulation in the skin had changed due to unknown reasons. Tatsuno et al. [19] found that the molecular mechanisms of TTX transfer and accumulation in the skin and liver are different, and that the accumulation rate of TTX in the skin will decrease with the increase in the dosage. Yosu-Yamhasita et al. [30] reported that a toxin binding protein (PSTBP) was involved in TTX transfer from the blood to the skin. When a large amount of TTX circulates in the blood, PSTBP could be saturated with TTX, leading to a decrease in the TTX accumulation rate in the skin. Therefore, it was speculated that when the PSTBP and TTX binding reached saturation, the TTX in the blood was no longer transported to the skin, resulting in the decrease in TTX concentration in the skin but the increase of TTX concentration in the blood. At the same time, TTX had been transferred to other tissues, including the gallbladder and kidney by other transporters, leading to the corresponding increase in toxin concentration.

In the present study, a dynamic model of TTX in *T. obscurus* was established to reflect the long-term elimination pattern. TTX content in the tissues showed a downward pattern, with elimination rate constant K from 0.12 to 8.06. Earlier TTX elimination experiments [20,23,26] were mostly carried out within short periods of time(60–168 h), mainly for observing TTX transfer, accumulation, and anatomic distribution. Ikeda et al. [26] studied TTX elimination in *T. rubripes* for 168 h after the IMA, and found that the toxin content showed a decreasing pattern of 8–12 h, then gradually increased from 24–168 h, and finally, about 89% accumulated in the skin. Wang et al. [23] used OGA for administration and found that TTX absorbed by the digestive tract was first transferred to the liver, and then to the skin, resulting in a pattern of decreasing and then increasing toxin content in the skin during the elimination stage. To observe the elimination stage after a long-term accumulation, the experiment was carried out for as long as 39 days (936 h). It was found that TTX content decreased gradually in all tissues during the whole elimination period, and there was not a notable increase in TTX in the skin tissue. This may be explained by the fact that the elimination stage was relatively long and short-term patterns could not be reflected. Unlike previous reports, a dynamic model was established in the present study to reflect the long-term elimination pattern. Analysis of the elimination process in nine tissues of *T. obscurus* showed that the process in vivo fit to a first-order elimination kinetic equation, similar to the Dyble kinetic equation for the elimination of microcystins [31]. It was possible to calculate the elimination rate constant and half-life. It was possible to predict that TTX in the skin of the high-dose group of the exposure group would be below the regulatory limit after elimination for 12.3 days. The kinetic model could be applied to a TTX level reduction control in pufferfish cultivation.

Seven kinds of TTX derivatives were found in different tissues of *T. obscurus*, but one of these, the anhydro-5,6,11-trideoxy TTX was only found in the skin. So far, more than 20 TTX structural analogs with different toxic potential have been found in pufferfish [3]. Jang et al. [32] reported that TTX and 5,6,11-trideoxyTTX were the major toxin components, whereas 4-epiTTX 4,9-anhydroTTX, 5-deoxyTTX, and 11-deoxyTTX were minor TTX analogs in the toxin profile of *Fugu niphobles*, *Tetraodon nigroviridis,* and *Tetradon biocellatus*. Recently, Tonol et al. [33] discovered new TTX analogs in Brazilian pufferfish tissues and microbiome through UPLC qTOF-MS/MS analysis in combination with molecular network techniques. Our study is the first to report the distribution of TTX analogs among skin, liver, blood, and another six tissues in *T. obscurus*, and the existence of five kinds of TTX derivatives. It should be noted that due to the lack of TTX derivative standards, only qualitative analyzes by UPLC-MS/MS of these derivatives were possible. The above findings indicate that TTX in the feed had indeed undergone biotransformation or degradation in *T. obscurus* being converted into a series of derivatives. Nevertheless, the mechanism of transformation needs to be further studied. Yang et al. [34] isolated a TTX-producing Aeromonas strain from the ovary of the pufferfish *Takifugu obscurus*. The possible effects of the strain on the TTX accumulation in the adult pufferfish or the existence of other toxin-producing strains in the target tissues should be considered. TTX and five other derivatives were also found in *T. niphobles* (current name: *T. alboplumbeus*) [32], but anhydro-5,6,11-trideoxy TTX was not detected. This toxin was previously detected in *Fugu poecilonotus* [35]. Nagashima et al. [36] found that non-toxic pufferfish species contained TTX derivatives with a lower toxic potential, and suggested that TTX could be biotransformed into other less toxic forms. Further studies on TTX derivatives in *T. obscurus* will help to identify the toxic potential of these derivatives.

## 4. Conclusions

This paper reports the first accumulation and elimination study of TTX in the pufferfish *T. obscurus.* A TTX exposure model of *T. obscurus* was established by feeding with wild *N. semiplicata* to simulate the natural food chain environment. The juvenile *T. obscurus* could accumulate TTX efficiently, and the distribution of TTX was tissue-specific. TTX content in the tissues showed a downward pattern, with an elimination rate constant K values ranging from 0.12 to 8.06. A Kinetic model to reflect the long-term elimination pattern of TTX in *T. obscurus* was established. This model could be applied to the control of TTX reduction during pufferfish cultivation in the future. The identification of seven TTX derivatives in different tissues of *T. obscurus* will be helpful to investigate the toxic potential of these derivatives.

## 5. Materials and Methods 

### 5.1. Materials

#### 5.1.1. Instruments and Reagents

Analytical instruments included an ACQUITY ultra-performance liquid chromatograph (UPLC, Waters, Miford, MA, USA), and a Xevo TQS quadrupole mass spectrometer (Waters, Manchester, UK), with an electrospray ion source. Other instruments included an MS2 Vortex Mixer (IKA, Wilmington, NC, USA); an Avanti JXN-30 refrigerated high-speed centrifuge (Beckman Coulter, Brea, CA, USA); an N-EVAP112 nitrogen evaporator (Organomation Associates, Berlin, MA, USA); a Visiprep SPE Vacuum Manifold (Supelco, Bellefonte, PA, USA); and an SK8200GT ultrasonic cleaner (Shanghai Kudos Ultrasonic Instrument Co., Ltd., Shanghai, China).

The TTX standard was purchased from Dr. Ehrenstorfer GmbH, Germany. Immuno-affinity columns (3 mL, 60 mg) were purchased from Jiangsu Meizheng Bio-Tech Inc., China. Milli-Q ultrapure water was used. Methanol, acetonitrile, acetic acid, and ammonium acetate were HPLC grade.

#### 5.1.2. Biological Materials

Two hundred three-month-old *T. obscurus* (20 ± 1 g, 4–5 cm) were purchased from the Shanghai Fisheries Research Institute. The protocol was approved by the Ethics Committee of the Marine Fishery Research Institute of Zhejiang on 5 May 2018 (Project identification code: 2017YFC1600701).

Fish formula feed contained 45% of crude protein, 4% of fat, and 15% of lysine. It was stored in a dry environment at room temperature.

Three batches of the sea snail *N. semiplicata* were collected from coastal areas of Xiangshan, Ningbo city in Zhejiang, from July to September 2018. and used as toxic feed. The soft tissue was taken out, cut into small pieces, thoroughly homogenized, packed in bags and stored at −18 °C until subsequent use. The TTX concentration in the aforementioned homogenates was determined by UPLC-MS/MS as 600 ng/g, 210 ng/g, and 100 ng/g, respectively. No TTX derivatives were detected in any of the *N. semiplicata* samples.

### 5.2. Culture Environment and Diet

*T. obscurus* was acclimated in 120 L tank, at a density of 30 fish per tank, and kept without feeding for 1 week in brackish water with salinity 8–10 and temperature of 22–25 °C, under a 12 h light:12 h dark photoperiod. Dissolved oxygen (>6 mg/L), ammonia nitrogen (<1 mg/L), and pH (7.5–8.0) conditions were maintained. After the acclimation period, *T. obscurus* was fed daily with fish feed (3% fish weight).

*T. obscurus* specimens were randomly divided into four groups: control group and low-, medium- and high-dose exposure groups, in which juvenile fish were fed with formula feed mixed with non-toxic shrimp (control), 100 ng TTX/g (low), 210 ng TTX/g (medium), and 600 ng TTX/g (high) dose exposure of TTX-containing *N. semiplicata* homogenate (*N. semiplicata* meat: formula feed = 1:2 wt).

**Figure 4:**
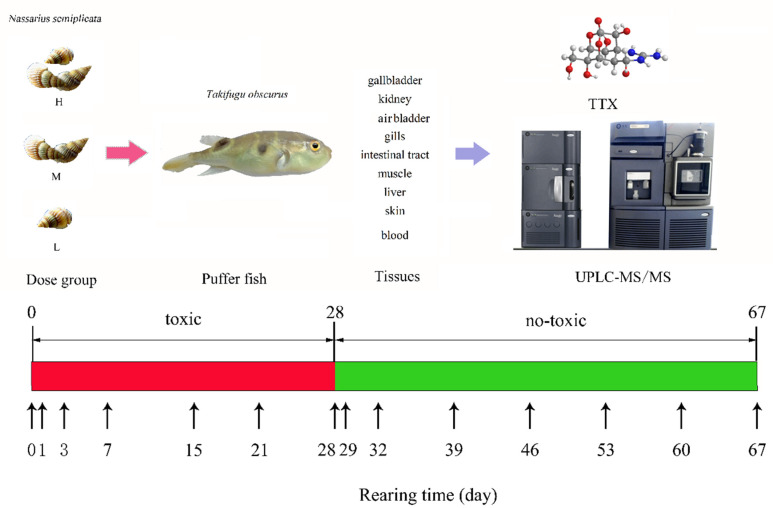
Diagram of the TTX accumulation and elimination experiment. The fish was fed with toxic feed until the 28th day, then fed with non-toxic feed until 67th day.

During the 28-d accumulation period, juveniles were fed the mixed feed daily (3% of their body weight). The unconsumed feed was removed after 0.5 h and weighed to calculate the actual daily intake. One-third of the tank water was renewed daily, and it was continuously filtered to maintain water quality. On day 28, the juveniles were transferred to new brackish water, and the feed for *T. obscurus* in the dose exposure groups was changed to the regular formula feed during the whole elimination period. The accumulation and elimination experiment schedule is shown in Figure 4. Three *T. obscurus* were randomly collected on days 0, 1, 3, 7, 15, 21, 28, 29, 32, 39, 46, 53, 60, and 67. Tissues including the gallbladder, kidney, air bladder, gills, intestinal tract, muscle, liver, skin, and blood were sampled and stored at −18 °C until analysis.

### 5.3. Analysis of TTX and Its Analogues

Quantitative detection of TTX was performed using the previously reported UPLC-MS/MS method [37]. Briefly, 10 mL of 1% acetic acid in methanol was added to an appropriate amount (0.5–2 g) of homogenized samples (skin, liver, muscle, etc.). The samples were vortexed for 3 min, sonicated at 60 °C for 15 min, cooled to room temperature, and centrifuged at 8000 rpm for 7 min. Five milliliters of the supernatant was diluted with 20 mL of PBS buffer, and the solution pH adjusted to 6.9–7.1 with 1 mol/L NaOH. Subsequently, the sample solution was loaded onto an immuno-affinity column, rinsed with 8 mL of 20% aqueous methanol solution, and finally eluted with 4 mL of 2% acetic acid in methanol. The obtained solution was concentrated with nitrogen at 60 °C and then dissolved in 5 mmol/L ammonium acetate/acetonitrile (V/V = 1/9) solution containing 0.1% formic acid to a final volume of 1 mL. Finally, the samples were filtered through a 0.22-μm filter prior to the analysis.

The chromatographic parameters were as follows: column, ACQUITY UPLC BEH amide (50 mm × 2.1 mm, 1.7 μm); sample chamber temperature, 4 °C; column temperature, 40 °C; injection volume, 10 μL; mobile phase A, 5 mmol/L ammonium acetate solution containing 0.1% formic acid, mobile phase B, acetonitrile; and flow rate, 0.3 mL/min. The gradient elution conditions were set as follows: 0–1.5 min, 10% A; 1.5–4 min, 60% A; 4–4.1 min, 60%A; and 4.1–5 min, 10% A.

The mass spectrometry parameters were as follows: electrospray ion source, positive ion (ESI+); mode, multiple reactions monitoring (MRM); capillary voltage, 3.0 kV; ion source temperature, 110 °C; desolventizing gas temperature, 350 °C; cone gas flow rate, 50 L/h; and desolventizing gas flow rate, 600 L/h. Data acquisition was conducted in MRM mode, and related parameters for TTX and its analogs are shown in Table 4.

### 5.4. Kinetic TTX Elimination Model

The elimination stage of TTX in *T. obscurus* was fit to a first-order loss kinetic model. The TTX concentration and elimination time were calculated by using the equation:

(1) $$C_{t}=C_{0}e^{-kt}$$

where *C_0_* and *C_t_* are the TTX concentration in the tissue (ng g^−1^) at time 0 (beginning) and time *t* (end of the elimination process), respectively, *t* is time (day), and *K* is the elimination rate constant (d^−1^) obtained from the equation:

(2) $$K=\left(\ln C_{0}-\ln C_{t}\right)/t$$

The half-life (*B*_1/2_) of TTX in each tissue was calculated based on the elimination rate constant *K,* as shown in Equation (3) [38]:

(3) $$B_{1/2}=\frac{\ln2}{K}$$

### 5.5. Statistical Analysis

The accumulation ratio of TTX was evaluated by comparing the actual intake and feeding amount at the end of the accumulation period. The accumulation rate was calculated by dividing the TTX concentration by accumulation days. The accumulation constant was calculated by dividing the TTX concentration in the tissue by feeding concentration. All experimental measurements were performed with at least three replicates for each data point. Elimination model regression analyses were performed according to the CurveExpert Professional software (version 2.6.5, Hyams Development, Chattanooga, TN, USA). *p*-values less than 0.05 were considered statistically significant. OriginPro software (version 9.6, OriginLab, Northampton, MA, USA) was performed for statistical analyses and scientific mapping, respectively.

## Figures and Tables

**Table 1:** Tetrodotoxin (TTX) accumulation ratio of whole *T. obscurus* in exposure dose groups *n* = 6.

Exposure Dose Group	TTX in Feed (ng/g)	Feed Intake (g)	Total TTX (ng)	Actual Accumulation of TTX (ng)	Accumulation Ratio (%)
Low-dose group	100	25.67 ± 2.1	2567 ± 210	1032 ± 104	40.20 ± 5.2
Medium-dose group	210	26.03 ± 3.2	5466 ± 320	1955 ± 110	35.76 ± 4.9
High-dose group	600	23.75 ± 2.4	14250 ± 240	5420 ± 173	38.03 ± 4.1

**Table 2:** Parameters of the TTX elimination kinetics in *Takifugu obscurus.*

Dose Group	Tissues	TTX-C_0_ Day 28 (ng/g)	TTX-C_t_ Day 67 (ng/g)	Elimination Rate K (d^−1^)	Half-Life Period B_1/2_ (d)
Low-dose group	gallbladder	87.96	9.53	0.084	8.25
	kidney	91.53	8.55	0.391	1.77
	air bladder	178.1	29.04	0.042	16.50
	gills	170.29	6.72	0.088	7.88
	intestinal tract	101.23	3.33	0.236	2.94
	muscle	10.68	2.32	0.040	9.16
	liver	262.18	4.42	0.387	1.79
	skin	717.34	135.66	0.042	16.50
	blood	189.35	14.42	0.105	6.60
Medium-dose group	gallbladder	238.84	19.79	0.132	5.25
	kidney	244.47	14.03	0.496	1.40
	air bladder	458.22	22.909	0.138	5.02
	gills	435.86	10.49	0.100	6.93
	intestinal tract	291.46	8.14	0.156	4.44
	muscle	20.51	2.79	0.056	12.38
	liver	427.62	27.88	0.399	1.74
	skin	1484.20	358.79	0.035	19.80
	blood	333.96	22.25	0.068	10.19
High-dose group	gallbladder	460	50.25	0.100	6.93
	kidney	486.47	29.07	0.320	2.17
	air bladder	757	43.86	0.098	7.07
	gills	650.12	54.82	0.075	9.24
	intestinal tract	622.93	18.1	0.12	5.78
	muscle	99.36	7.4	0.092	7.53
	liver	1840.3	41.18	0.952	0.73
	skin	6000	699.35	0.076	9.12
	blood	750.13	50.1	0.059	11.75

**Table 3:** TTX and its derivatives found in *Takifugu obscurus.*

TTX Analogues	*Takifugu obscurus* Tissues								
	Gallbladder	Kidney	Air Bladder	Gills	Intestinal Tract	Muscle	Liver	Skin	Blood
TTX	√	√	√	√	√	√	√	√	√
5-deoxy TTX	√	√	√	√	√	√	√	√	√
11-deoxy TTX	√	√	√	√	√	√	√	√	√
4,9-anhydro TTX	√	√	√	√	√	/	√	√	√
6,11-dideoxy TTX	/	√	√	√	√	√	√	√	√
5,6,11-trideoxy TTX	√	/	√	√	√	/	√	√	√
anhydro-5,6,11-trideoxy TTX	/	/	/	/	/	/	/	√	/

√ means present, / means not detected.

**Table 4:** Parameters of the UPLC-MS/MS system to measure TTX and analogs.

Analyte	Retention Time(min)	Precursor Ion(m/z)	Product Ion(m/z)	Cone Voltage (V)	Collision Energy(eV)
TTX	2.77	320.0	302.0 * 161.8	45	25 35
5-deoxy TTX/ 11-deoxy TTX	2.61	304.5	286.29 162.10	40	25 20
4,9-anhydro TTX	2.61	302.20	256.13 161.83	40	35 25
6,11-dideoxy TTX	2.51	288.54	270.11 224.69	40	25 25
5,6,11-trideoxy TTX	2.22	271.90	254.11 162.07	40	25 20
anhydro-5,6,11-trideoxy TTX	1.85	254.02	161.98	40	25

*: Quantitative ion.
